# Trained Eyes: Experience Promotes Adaptive Gaze Control in Dynamic and Uncertain Visual Environments

**DOI:** 10.1371/journal.pone.0071371

**Published:** 2013-08-12

**Authors:** Shuichiro Taya, David Windridge, Magda Osman

**Affiliations:** 1 Department of Human Sciences, Taisho University, Tokyo, Japan; 2 Centre for Vision Speech and Signal Processing, University of Surrey, Guildford, United Kingdom; 3 School of Biological and Chemical Science, Queen Mary College, University of London, London, United Kingdom; University of California, Davis, United States of America

## Abstract

Current eye-tracking research suggests that our eyes make anticipatory movements to a location that is relevant for a forthcoming task. Moreover, there is evidence to suggest that with more practice anticipatory gaze control can improve. However, these findings are largely limited to situations where participants are actively engaged in a task. We ask: does experience modulate anticipative gaze control while passively observing a visual scene? To tackle this we tested people with varying degrees of experience of tennis, in order to uncover potential associations between experience and eye movement behaviour while they watched tennis videos. The number, size, and accuracy of saccades (rapid eye-movements) made around ‘events,’ which is critical for the scene context (i.e. hit and bounce) were analysed. Overall, we found that experience improved anticipatory eye-movements while watching tennis clips. In general, those with extensive experience showed greater accuracy of saccades to upcoming event locations; this was particularly prevalent for events in the scene that carried high uncertainty (i.e. ball bounces). The results indicate that, even when passively observing, our gaze control system utilizes prior relevant knowledge in order to anticipate upcoming uncertain event locations.

## Introduction

Imagine that a friend, who happens to be a fan of tennis, has taken us out to watch a live sports match, but we are unfamiliar with the game. How do we organize our eye-movements so we attend to the changing relevant aspects of the game? More to the point, is our eye-movements different to that of our experienced friend? In order to begin answering these questions, we need to take into account the various limitations in our visual system.

For a start, one of the fundamental limitations is that our eyes have the highest resolution approximately within 1 degree visual angle around the centre of gaze (i.e. fovea). In addition to our limited visual accuracy, visual information gain is usually achieved from locations corresponding with the centre of gaze [1]–[3]. In dynamic scenes, one key issue is that the location of relevant information often changes from one moment to the next. Thus we need to reorient our eyes as quickly as possible toward the location of transient ‘events’ in order to acquire information which is critical to understanding what is going on in visual environments. The problem then is that eye-movements directed towards dynamic events often require big leaps from one gaze location to another. The rapid and distant eye movements also have to be successional, especially when a target that we are looking for is moving rapidly and continuously. While in such situations saccades help to efficiently redirect our gaze, it is also well known that saccades also suppress visual information processing (i.e. *saccadic suppression*, e.g. [4]). Because of active suppression of information processing, saccades hamper information gain from the dynamic scene. Thus our gaze control system needs to be strategic in order to maximise information acquisition and to minimise information loss, both of which are affected by saccades.

One methodology that has been successful in making headway in the direction of understanding gaze control is eye-tracking research. Eye-tracking in a dynamic environment is important because our gaze allocation is tightly coupled with the location of a target and the timing of the target, i.e. when it is required for an on-going task [5]–[12]. To illustrate, in one study, participant’s eye-movements were measured while conducting their daily routine tasks (e.g. tea making) [8]. It was found that participants employed a gaze control strategy designed to conserve limited cognitive resources by only looking at the task-relevant object (e.g. teapot) just before they needed to use it. Of course, such a “just in time” strategy [5] may not be effective unless prior knowledge of the routine is known. In addition, previous studies have shown that adaptive gaze control is influenced by the observers’ level of expertise of the task [13]–[18]. Thus, timing in the choice of eye-movements we make is important and suggests that, the level of knowledge we possess about a scene, as well as what we can learn about what to look at in a scene, and when to look, helps to conserve valuable cognitive resources.

Although previous studies have reported that past experience modulates eye-movement control, less is known about the effect of experience on the eye-movement of observers who are merely watching the other’s activity [18]–[20]. Fewer still have examined the role of prior experience in such situations. Therefore, in this study, we consider the role of prior experience on anticipatory eye-movement behaviour while observing the activity of another. In the present study we addressed three questions concerning adaptive gaze control in dynamic environments, and we examine them using a dynamic natural visual scene. The first question is: how do observers deal with the trade-off inherent in saccadic eye-movements in dynamic scenes? To address this, we recorded and analysed eye-movements of participants while they were watching a short video clip of a tennis game. Tennis videos are ideal visual stimuli for our purpose. This is because viewers watching tennis matches are naturally required to track the location of the ball in order to comprehend the critical events occurring in the game (e.g. which player is going to be awarded a point?). Also, because the ball location is continuously and rapidly changing its location, observers must also make saccades actively to follow the change in ball location. Therefore, measuring eye-movements during the observation of a tennis match provides us an opportunity to study when and how saccades are promoted/inhibited in complex dynamic scenes for visual information acquisition.

The second question we addressed is: how does prior experience of the dynamic scenes modulates our gaze allocation strategies? To this end, we put to the test three predictions about the effect of experience on saccadic eye-movements. First, if experience modulates eye-movements then observers with rich experience of the game of tennis (i.e. knowledge of the rules and prior experience in playing the game) should make saccades with higher accuracy than those with less experience. That is, experienced observer should bring their eyes closer to the upcoming event location in a dynamic scene as compared to observers with less experience. Second, experienced observers should be able to anticipate the upcoming event location faster than less-experienced observers, and this should lead to earlier saccade onset. Third, when compared with less-experienced observers, experienced observers should maximize the information acquisition and minimize the information loss more efficiently - both of which are thought to be caused by saccades. More specifically, to accomplish effective information acquisition, experienced observers should make fewer saccades and/or shorter amplitude of saccades to orientate their eyes to the next relevant event location. We expected that experienced observers would predict the upcoming event location more precisely than less-experienced observers, thus their saccade should take the shortest distance from their current location to the next event. On the other hand, less-experienced observers should not be able to accurately predict the upcoming event location, thus their saccades should lead to larger distances from their current location to the next relevant event. To compensate for the eye-movement error less-experienced observers should make more saccades to bring their eyes closer to the event location. We predict that, experienced observers should also make successful smooth pursuit eye-movements when tracking the ball (e.g. [12], [15]), thus they should direct their eyes to the event location while making fewer and shorter saccades compared to less-experienced observers.

The third question we considered is: does experience of the visual scene help effective gaze allocation strategies when faced with uncertainty of visual events? Specifically we investigated how the accuracy of gaze control is influenced by the uncertainty arising from when and where the events we should look at occur. To this end, saccades were recorded around two types of ball events, hits and bounces. Ball bounces are important because their location is critical for the allocation of a point. Ball hits are important because the racket angle and speed of impact decides where and when the ball bounces next. The location of a hit is relatively easier to predict because the location of a player who approaches the ball with their racket is a strong visible predictive cue as to where the next ball hit will be made. In contrast, predicting the location of ball bounce is much more difficult because observers need to anticipate the ball location based on the angle or speed of the ball at the moment of a preceding hit. We hypothesise that there is higher uncertainty attached to ball bounce events as compared to ball hits, because there are stronger predictive cues for ball hits than bounces. Therefore, eye-movement control should reflect an interaction between uncertainty of the ball event (i.e. bounce, hit) and the experience of observer.

## Materials and Methods

### Ethics Statement

The experiment was approved by the local ethics committee of the University of Surrey. Written informed consent was obtained from each observer prior to the experiment.

### Observers

Forty volunteers took part in the experiment (mean ± SD = 22.7±4.6 years old, range 19–45 years).

Before the main experiment began, participants answered a questionnaire regarding their knowledge and experience of tennis and other racket sports. We asked seven questions relating to the rules of tennis, to which responses were recorded as either yes or no (‘rule questions’, Table 1). Participants were also asked to answer questions related to racket sports they played in the last five years, they chose from seven sports: again the response format was the same as the rule questions (‘exercise question’, Table 2). For both sets of questions we adopted a simple scoring system. We simply assigned 1 point to each ‘yes’ response to the rule questions, and assigned 1 point to each sport the observers played in the last five years reported in the exercise question. The mean of points based on an aggregate of responses to both rule and exercise question was 4.43 (SD = 2.3, range 0–7) and 3.5 (SD = 1.3, range 0–5), respectively. We used the sum of the points assigned to each of the two questions as an index of observers’ experience, and tested whether observers experience score was correlated with eye-movement measures.

**Table 1:** Questions about tennis rules.

a)	I can accurately judge how an individual point has been awarded
b)	I know what the scoring system is (e.g., game, set, match)
c)	I know what a ‘love’ set refers to in a game of tennis
d)	I know what ‘let’ refers to in a game of tennis
e)	I know what ‘foot fault’ refers to in a game of tennis
f)	I know what a ‘rally’ refers to in a game of tennis
g)	I know what a ‘tiebreaker’ refers to in a game of tennis

**Table 2:** Question about racket sports experience.

“Which of the following do you/have you played in the last five years (Please select from the options below. You can select more than one option).”	
a)	Squash
b)	Badminton
c)	Soft ball
d)	Racquet
e)	Mtkok (Paddle ball)
f)	Soft tennis
g)	Tennis

### Experimental Design and Stimuli

The experiment consisted of four blocks. Ten short video clips of a singles tennis match were used as visual stimuli (25 Hz, 720 × 576 pixels/frame, mean ± SD = 8.8±1.9 s, with audio). Each clip was presented once in each block but presentation order was randomized. The clips were displayed on a 19 inch colour CRT monitor at a 60 cm viewing distance. A clip subtended 32.7 × 24.8 deg in visual angle on the monitor. Stimulus presentation and data acquisition were controlled by SR Research Experimental Builder running on a PC.

All clips were selected and extracted from a commercial DVD of a singles tennis match (1993 Wimbledon Championships ladies’ single). We only included clips of ‘play shots’ of games in which a camera faced down the whole tennis court from behind the centre mark. Each clip began from just before a service and ended after a point was clearly decided. Camera edits (scene cuts) were not included in any of the selected clips because they could have significant effects on eye-movement control (e.g. [22]–[24]). Neither interpolated closed shots nor replays were included in any of the selected clips.

To encourage observers to pay attention to the display during the whole duration of a clip, they were to report the precise ordering of where their attention was directed towards during the observation of the clip. Thus, in each trial, after a clip was presented, a new screen appeared in which two list boxes were presented on the left-side and the right-side of the screen. The items listed in the left box were Player A (top of the screen), Player B (bottom of the screen), Ball, Net, Horizontal line, Vertical line, Ball boy/girl, Audience, and Umpire. The initial ordering of these items was randomized for each trial. A single mouse click moved each item from the left box to the right box (or vice versa), and the moved item was placed from top to bottom in the box.

### Eye Movement Recording

Observer’s eye movements were recorded while they were watching the tennis clips. An infra-red video-based eye-tracker sampling at 1000 Hz (Eyelink 1000, SR Research) was used for eye tracking. Viewing was binocular, but only the left eye was tracked. A chin-and-forehead rest was used to stabilize participant’s head. At the start of experiment calibration and validation were performed using a series of nine dots arranged in a square grid. In addition, at the start of each trial a bull’s eye was presented at the centre of the screen. Participants were asked to fixate on this fixation marker and if the deviation between the measured eye position and the fixation maker was too large (>1.5 degree in visual angle) a recalibration was conducted.

An SR Research saccade parsing algorithm was used on the original 1000 Hz raw data to identify saccades with a combination of 50°/s velocity threshold and an 8,000°/s^2^ acceleration threshold. All saccades which ended outside of the screen were excluded. The mean and median of saccade duration were 47.7 ms and 37.0 ms, respectively (±49.6 SD). The difference between the mean and median values indicates the skewed distribution of saccadic durations.

### Analysed Saccadic Measures

The goal of this study was to clarify how our visual system adaptively handles the trade-off regarding saccade control (information acquisition and information loss, both of which could be caused by saccades). In addition, this study also examines how experience modulates adaptive saccade control behaviour. To explore the three questions we posed, we analysed five measures of saccades, *proximity*, *error*, *onset*, *frequency*, and *amplitudes*, all of which were based on the saccades made around the ball events (i.e. hit and bounce).

There were 32 hits and 39 bounces in the 10 selected stimulus clips. Because we wanted to see the time course of the saccades around a ball event, it was important to avoid the temporal overlap of the ‘target event’ (which is the target of analysis) and the other residual events (which occur either before or after the target event and were not the target of analysis). In the stimulus clips, the residual events were especially frequent in the −400 ms to 0 ms from the time the target hit occurred and in the 0 ms to 400 ms from the time the target bounce occurred (see Figure S1). For this reason we excluded all pairs of successive events of inter-event interval that were less-or-equal to 400 ms (e.g. if a bounce occurred 320 ms after a hit, both the bounce and the hit were excluded from the analysis). After this manipulation 11 hits and 17 bounces were used as the target events (since the 10 selected clips were presented four times to each subject the whole dataset consists of four times these 11 hits and 17 bounces). The mean of hit-to-bounce interval, bounce-to-hit interval, and hit-to-hit interval (e.g. volleys and smashes; the cases where no bounces occurred between successive hits) were 871, 515, and 900 ms, respectively.

**Figure 1:**
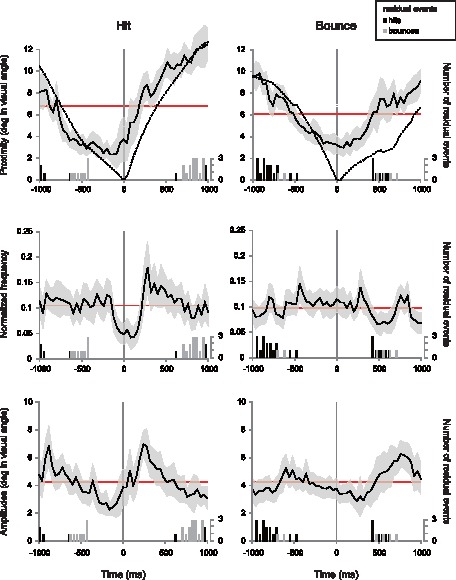
Eye-movement measures plotted as a function of time. Top panels: the average distance between saccade-end-points and target event location (solid lines) and the average ball-event distance (dashed lines). Middle panels: the normalized frequency (number of saccades). Bottom panels: the average saccadic amplitudes. The data in left panels consist of saccades measured around target hits and the data in right panels consist of saccades measures around target bounces. The gray-shaded area indicates Bonferroni corrected 95% confident intervals corrected; i.e. the confidence interval was set at the level of 1–0.05/51 (number of bins). The columns show how many residual events (i.e. the events which were not the target of analysis) were included in the period ±1000 ms around each target event (right ordinate).

*Proximity* refers to the average distance between the location where saccades landed (hereafter, saccade-end-point, SEP) and the location where the target event occurred. This measure gives an indication of how quick and near the eyes relocated toward the event location. For example, when the target hit occurred at time *T* (ms, calculated by 40 times multiplying the frame number; e.g., if a target hit occurred at the 10th frame in a video clip, *T* was 400 ms), all of the saccades which ended at *T*±1000 ms were pooled. Then the distance between each SEP (*X_SEP_*, *Y_SEP_*) and the target event location at the time T (*X_EVENT_*, *Y_EVENT_*) was calculated for each pooled saccade in the *T*±1000 ms epoch (i.e. square root of (*X_SEP_*-*X_EVENT_*)^2^+(*Y_SEP_*-*Y_EVENT_*)^2^). This calculation was repeated for all target events (11 hits and 17 bounces) for each observer. Then the calculated distance (*proximity*) was assigned to one of 51 40 ms bins (target event frame ±25 frames). We averaged each of the 51 bins, which provide a time course of *proximity* for each subject. Then finally we averaged 51 bins for 40 observers (Figure 1 top panels).

*Frequency* and *amplitudes* refer to the average number of saccades (normalized by the number of events × trials) and average saccadic amplitudes ended in each frame. These measures were calculated across *T*±1000 ms epochs using a similar procedure used to calculate *proximity*. That is, we counted the number of saccades and calculated the average saccade amplitude in each of the 51 bins (*T*±1000 ms around each event; Figure 1 middle and lower panels, respectively). These measures enable us to understand the eye-movement strategy that our visual system uses in a dynamic and uncertain visual environment. Recall that making a saccade means accepting a risk in loss of critical information, because visual information processing is considerably degraded during saccadic eye-movements. To handle this trade-off, we expected that, the visual system should reduce the saccadic amplitudes, or else reduce the number of saccades temporally around an informative event.

In addition, as sub-measures of *proximity*, we also analysed *error* and *onset*. *Error* is the minimum distance between SEP and the target event location. Specifically, we pooled the saccade that ended between *T*-400 to *T* ms and tagged the one which marked the shortest *proximity* (SEP-event distance) as *error*. By selectively analysing the saccades made in this period we avoided including more than one event in the epoch to be analysed. *Onset* is the time stamp of the start of the saccade that marked as *error*. These measures were used to see if experience could improve the precision of the saccade and if experience could change the timing of saccade onsets.

## Results

Figure 1 shows the time course of *proximity*, *frequency*, and *amplitude* (from top to bottom), all of which were plotted as a function of changes in time, specifically, ±1000 ms (25 frames) from the time where an event occurred. The left and right panels show the measure computed around hits and bounces, respectively. The shaded area in each panel indicates Bonferroni corrected 95% confident intervals, where the confidence interval was adjusted by the number of bins (i.e. the confidence interval was set at the level of 1–0.05/51). The horizontal line in each panel indicates the mean of the eye-movement measure averaged across ±1000 ms around each event. Thus if the shaded area and the horizontal line did not overlap, this indicates that the measure was different (either higher or lower) than the average value at that epoch with a significance level of 0.05. The columns in Figure 1 show how many residual events (i.e. hits and bounces occurred either before or after the target event) were included in the period ±1000 ms around each target event (right ordinate).

### How Saccades are Made Around Dynamic Events?

In the graphs of *proximity* (Figure 1 top panels) the mean distance between a ball and a target event location in each time is also plotted as a function of time (dashed lines). The figures reveal the anticipatory nature of saccades in dynamic scenes; the SEPs are closer to the target event location than the ball location before the event occurs. This eye-movement pattern replicates previous studies that have recorded eye-movements in a video game, in which observers were required to follow a moving ball (‘breakout’, [12]). The *proximity* value at the lower peak (the smallest value of proximity in the 51 bins) is slightly smaller for hits than bounces, suggesting that higher uncertainty associated with bounces deteriorated eye-movement control. This was supported by the results of a paired *t*-test conducted with the *proximity* value at lower peak across hits and bounces (*t*_39_ = 7.66, *p*<.001). On average the lower peak was 0.93 (SD = 0.41) deg (in visual angle) for hits and 1.61 (SD = 0.57) deg for bounces (Note that the lower peak is different from *error* that is the minimum value of *proximity* in the period 400 ms before each event). In addition, the higher uncertainty associated with bounces as compared with hits is also expressed in the time when proximity marked the lower peak. The average *proximity* reached a lower peak before a hit, whereas it reached a lower peak after a bounce (*t*_39_ = 3.72, *p*<.001). On average the lower peak was 92 (SD = 243) ms *before* hits and 128 (SD = 247) ms *after* bounces.

The middle and lower panels in Figure 1 shows the *frequency* and *amplitudes* plotted as a function time, respectively. In the case of hits the number of saccades sharply decreased before a target hit was made and quickly increased after a target hit was made (Figure 1 middle left). The amplitude of saccades also gradually decreased before a target hit and quickly increased after a target hit (Figure 1 lower left). The patterns of saccadic eye-movements were as predicted; before a target hit, observers made saccades less frequently and with shorter amplitudes. It is possible that the visual system avoids information loss at the moment critical for predicting the next ball location by using this type of strategy.

The numbers and amplitudes of saccades around a target bounce reflect higher difficulty in predicting the bounce location as compared with the hit location (middle-right and bottom-right panels in Figure 1). The number of saccades was relatively constant around the moment of a target bounce. The reduction of frequency of saccades after the target bounce might be caused by the residual hits that occurred recurrently around 500 ms after the target bounce (black columns). These results suggest that most observers could not anticipate the timing and location of bounces, and therefore indicating that bounces were particularly hard to predict.

### Effect of Experience on Eye-movement Measures

**Figure 2:**
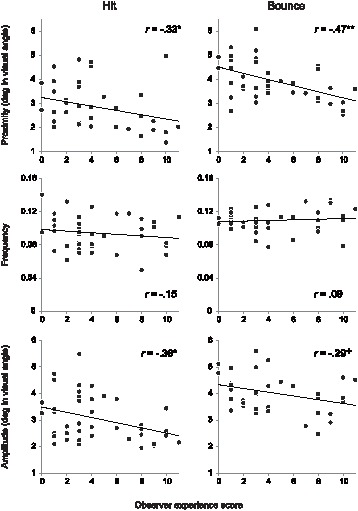
Scatter plot between the observer experience score and *proximity* (top panels), between the observer experience score and normalized *frequency* (middle panels), and between the observer experience score and *amplitude* (bottom panels). The left panels are the plots measured around the target hits and the right panels are the plots measured around the target bounces. Asterisks show the *p*-values for the correlation coefficients (^+^ *p*<.1, *p<.05, **p<.001).

**Figure 3:**
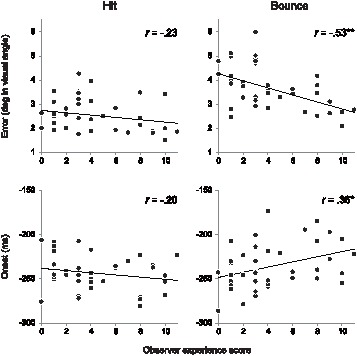
Scatter plot between the observer experience score and *error* (top panels), and between the observer experience score and *onset* (bottom panels). The left panels are the plots measured around target hits and the right panels are the plots measured around ball bounces. Asterisks show the *p*-values for the correlation coefficients (*p<.05, **p<.001).

To assess the effect of experience on saccade control, we calculated the correlation coefficient between observer experience score and each of the five eye-movement measures described above. To avoid the event overlap we calculated the individual average of the measure for each observer by collapsing across −400 ms to 0 ms from the time target event occurred. The correlation coefficients (Pearson’s *r*) were then calculated between the value of each measure and the observer experience score. The scatter plots between the observer experience score and each of the five eye movement measures are shown in Figure 2 (for *proximity*, *frequency*, and *amplitudes*) and Figure 3 (for *error*, and *onset*), with the correlation coefficients and the *p* values for them. We found that there were significant negative correlations between observer experience score and *proximity*, around hits and bounces. The negative correlation between observer experience score and *amplitudes* in the saccades measured around hits was significant, while for bounces the correlation was marginally significant. There was no significant correlation between experience score and *frequency*. In total, the negative correlation coefficients for *proximity and amplitude* suggest that experienced observers were able to locate their eyes closer to the event location by making shorter saccades as compared to less-experienced observers. The effect of experience on *proximity* was significant for both hits and bounces despite the difference in uncertainty for both types of ball events. However, the interaction between observer experience and differences in event uncertainty was found in *error* as well as *onset* (Figure 3); there was a significant negative correlation between observer experience score and *error* around target bounces, but not around target hits. We found a significant positive correlation between observer experience score and *onset* for target bounces but not for target hits. Taken together, these results suggest that observers’ experience with the dynamic scene modulates eye-movement control especially when the upcoming event has high uncertainty attached to it.

### Attention Allocation

**Figure 4:**
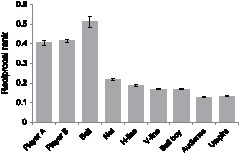
The results of the subjective attention allocation task. Player A is the player in the top of the display and Player B is the player in the bottom of the display. Note that the ordinate shows the reciprocal of the averaged rank (i.e. 1 means most attended and 9 means least attended), thus the items which were attended more get higher value. Error bars show ±1 SEM.

Figure 4 shows the results from observers’ responses to the task of reporting the order of their attention to the objects of interest in the scene. Note that in this figure the ordinate shows the reciprocal of the averaged rank (i.e. 1 means most attended and 9 means least attended), thus the items which were attended more get higher values. As shown in this figure, subjectively observers reported that they attended more to the ball and the players than the other items. Even though this ranking task could interfere with the typical eye-movement strategy of observers, as we have shown so far, the effects of prior experience are robust enough to overcome such residual factors. In addition, our previous results suggest that this kind of non-goal oriented task has little effect on eye-movement behaviours [25].

## Discussion

The general aim of this study was to examine in detail adaptive saccade control using a natural dynamic scene with associated uncertainties around key events. Our results clearly revealed that observers adopt adaptive strategies when making saccades in a natural dynamic scene even if the observers did not engage in the activity carried out in the visual scene. During the observation of tennis clips observers oriented their eyes toward the location of informative events (hit and bounce) utilizing saccadic eye movements (Figure 1). This kind of anticipatory gaze allocation is consistent with eye-movement patterns in players of ball sports and players of computer games [12], [14], [15]. Anticipatory eye-movements are imperative in a dynamic scene, especially when target events contain information for understanding what is going on in the scene (e.g. situation of ball sports). More importantly, our observers reduced the number and amplitudes of saccades around the events. In so doing, they were able to minimize the information loss which results from saccadic suppression.

The most notable finding in the current study is that prior experience of the context of the dynamic scene, in this case general familiarity with the rules and experience of playing tennis, improved eye-movement behaviour during mere observation of the game. We made three predictions about the effect of experience on saccadic control, namely experienced observers (as indexed by responses to questions concerning degree of procedural and declarative knowledge of the game) would show: (1) more accurate saccade control, (2) earlier saccade onset, and (3) fewer number and/or shorter saccades than less-experienced observers. In support of our predictions, we found that experienced observers could redirect their eyes more closely to the location of an upcoming ball event as compared to less-experienced observers. They achieved this by making shorter amplitudes of saccades. On the other hand, contrary to our prediction, the saccade began closer in time to the ball events for experienced observers than less-experienced observers.

Our results also demonstrated that the level of uncertainty of upcoming events modulated peoples’ gaze control strategy. Observers systematically reduced the number and amplitude of saccades around ball hits, and eye-event distance (*proximity*) peaked before the occurrence of a hit. On the other hand, observers did not reduce the number and amplitude of saccades around bounces and eye-event distance did not peak until the occurrence of the bounce. The difference in eye movement behaviour between these two events might be caused by the greater difficulty in predicting bounce location than hit location. Part of the difficulty of predicting bounce locations rests on the problems with inferring a three-dimensional trajectory of a ball from a 2D projection on the computer display. For ball bounces, observers have to predict the bounce location only with the motion of a ball (and possibly its shadow on the floor), which makes predictions difficult. On the other hand, when predicting hit location, two objects (racket and ball) approach each other thus the hit location can be estimated even without three-dimensional reconstruction. The analysis regarding experience revealed that prior experience has an advantage in facilitating predictions of the location of highly uncertain events. Experienced observers could bring their eyes much closer to the location of bounce than less-experienced observers. Smaller *error* (minimum SEP-event distance before an event) for bounces in experienced observers may also support the view that experience could help in making accurate predictions for uncertain events. Given that predicting the location of a hit is easier, both less-experienced observers and experienced observers could bring their eyes closer to the hit location.

Another critical difference between hits and bounces is the transversal component of ball trajectory. On bounces, the ball typically continues its movement in the horizontal direction, while inverting its vertical velocity. To follow the ball before and after the bounce observers need to move their gaze in transversal direction. On the other hand, on hits, the ball passes very close to where it came from, allowing the eyes to stay in a pre-hit location and catch the ball in its way back. This distinction may also account for the difference in frequency and amplitude between around target hits and around target bounces.

Evidence shows that people combine smooth pursuit with saccadic eye-movements in order to improve tracking of a moving object [12], [15], [21]. Our results also suggest that smooth pursuit may be involved while watching tennis videos. We hypothesize that by bringing their eyes closer to the event location through successful pursuit of the ball, observers can make fewer saccades. This hypothesis may explain the significant positive correlation between observers’ prior experience and *onset* around a bounce. Experienced observers may have successfully made smooth pursuits of the ball, which is why they made shorter saccades, bringing their eyes moderately close to the event location. This seems like a more efficient and reliable method, than making large saccades from a distant point in the visual scene.

Previous studies have examined the effect of experience on anticipatory gaze control in action observation [19], [20]. In these studies observers were asked to perform a block stacking task and observe an actor performing the same task. In both cases, observers showed predictive gaze shifts. In such situations what observers saw when they conducted the task and when they observed the actor’s action were quite compatible, thus direct matching between their own action and actor’s action is effective enough for the predictive gaze control. It is worth noting that in the current study, rule-based knowledge and prior experience of other racket sports does not directly map onto the events observed in tennis clips. So this type of knowledge could not be used directly to predict the forthcoming event locations which were projected on the computer screen. Crucially, in this study, what this implies is that people have a generalized predictive system which is supported by accumulated knowledge and prior experience of various relevant and related contexts to the observed scene.

In conclusion, we here found that observers make anticipatory eye-movements even when they are not directly engaged actively in the scene they are observing. In addition, we revealed that task-relevant experience helps gaze control in action observation. In combination with the event-related analyses we adopted, our measures of experience while simple were able to provide robust findings suggesting a clear distinction between saccadic control of observers with extensive experience compared with limited experience in tennis. Overall, this study demonstrated that our gaze control is adaptive, and can be facilitated by prior experience that is specifically related to the activity being observed [26].

## Supporting Information

Figure S1 **Histogram of residual events.** Here the number of residual events (i.e. the events which were not the target of analysis) included in the period ±1000 ms around the target event were plotted as a function of time. The left (right) panel shows how many residual hits and bounces occurred ±1000 ms around a target hit (target bounce) in the 10 video clips used in the experiment. We excluded the residual events ±400 ms from the target event (shaded area around 0 in the abscissa) from all of the analyses.
(EPS)
